# Nitrogen and Phosphorus Stoichiometry of *Bolboschoenus planiculmis* Plants in Soda–Alkali Wetlands Undergoing Agricultural Drainage Water Input in a Semi-Arid Region

**DOI:** 10.3390/plants14050787

**Published:** 2025-03-04

**Authors:** Yu An, Le Wang, Bo Liu, Haitao Wu, Shouzheng Tong

**Affiliations:** Key Laboratory of Wetland Ecology and Environment, Northeast Institute of Geography and Agroecology, Chinese Academy of Sciences, Changchun 130102, China; anyu@iga.ac.cn (Y.A.); wangle@iga.ac.cn (L.W.); wuhaitao@iga.ac.cn (H.W.); tongshouzheng@iga.ac.cn (S.T.)

**Keywords:** nutrient allocation, water environment, ecological adaptability, soda–alkali wetlands, plant growth

## Abstract

In semi-arid regions, wetlands often face water scarcity, salinity, and alkalinity stresses. Agricultural drainage water has been used to restore degraded wetlands, but it alters water quality and plant growth and resource distribution. Nitrogen (N) and phosphorus (P) stoichiometry reflect plant resource strategies. In China’s Songnen Plain, *Bolboschoenus planiculmis*, a key plant in soda–alkali wetlands and food for the rare white crane (*Grus leucogeranus*), is impacted by agricultural water input. However, the N and P stoichiometry in *B. planiculmis* and the influencing water variables remain unclear. This study analyzed N and P contents in *B. planiculmis* leaves, stems, tubers, and roots, and water variables. Results showed that leaf N content was highest, while tuber P content exceeded that of other organs. Leaf nitrogen to phosphorus (N:P) ratio was highest, and tuber’s was the lowest. N and P contents in plants were positively correlated, except between roots and stems. Redundancy analysis (RDA) revealed water temperature (T), oxidation-reduction potential (ORP), N contents, and water depth (WD) as key factors influencing N and P stoichiometry. Structural equation models (SEMs) indicated water T negatively affected plant N, while water nitrate nitrogen positively affected it. Water P content directly influenced leaf and stem P, and ammonium nitrogen affected aboveground P accumulation. Water T and WD directly impacted N:P ratios. These findings show that while agricultural drainage water alleviated aridification and salinization in degraded soda–alkali wetlands, exogenous N and P inputs significantly affected vegetation’s N and P utilization strategies.

## 1. Introduction

Nitrogen (N) and phosphorus (P) are crucial elements in ecosystems, influencing plant growth, energy conversion, metabolism, and stress resistance [1]. N serves as a fundamental constituent of chlorophyll, playing a pivotal role in plant physiology. Elevated N content significantly promotes chlorophyll biosynthesis, consequently enhancing photosynthetic efficiency and facilitating increased energy production for optimal plant growth and development [2]. N deficiency severely impairs plant metabolic processes, primarily by limiting the biosynthesis of essential proteins and chlorophyll pigments. Consequently, leaves turn yellow, and plants become stunted, ultimately resulting in significantly compromised plant vigor and productivity [3]. P significantly enhances root system development by stimulating root growth and branching, thereby improving the plant’s capacity for water and nutrient uptake [4]. Plants with low levels of N and P are more vulnerable to adversity [5]. The nitrogen to phosphorus (N:P) ratio is an important indicator of nutrient limitation in terrestrial ecosystems [6]. It can directly reflect the absorption and utilization of N and P by plants. When the ratio of N:P falls within the optimal range, it signifies balanced nutrient acquisition during plant growth, reflecting favorable nutritional conditions for optimal physiological development [7]. The N:P ratio in plant organs not only reflects the nutrient status of the plant itself but is also closely related to nutrient cycling in the ecosystem. Plants with a high N:P ratio may release more N during decomposition, thus affecting the N:P balance in the soil [8]. Consequently, investigating N and P stoichiometry of wetland plants provides critical insights into nutrient cycling processes and mechanisms within wetland ecosystems under global climate change scenarios.

Hydrological regimes in wetlands fundamentally govern vegetation composition and spatial distribution patterns [9,10]. Hydrological alterations significantly affect nutrient dynamics and vegetation stability in wetland ecosystems [11,12]. In recent years, the hydrology of natural wetlands has been increasingly impacted by drainage, ditching, and reclamation for agricultural development projects [13]. The inflow of agricultural drainage water can meet the water requirements of wetlands but also alters the water quality. Agricultural runoff typically carries a large amount of nutrients such as N and P. The influx of nutrients into wetlands alters aquatic nutrient concentrations and may infiltrate soil systems, modifying soil physicochemical properties and subsequently affecting plant growth dynamics and spatial distribution patterns [14]. Moreover, a large quantity of pesticides and fertilizers frequently used in farmland may be transferred to wetlands and cause pollution to water environments [15,16]. In the face of pollution stress, plants often adapt to the environment by adjusting their nutrient absorption and utilization strategies, ultimately affecting vegetation distribution and community structure, as well as population growth and reproduction [17]. Consequently, investigating N and P stoichiometry in wetland plants is crucial for understanding ecological adaptation mechanisms and optimizing water management under persistent anthropogenic influences, particularly agricultural drainage water input.

The soda–alkali wetland in the Songnen Plain, China, is of great significance for maintaining biodiversity, protecting the habitat of rare waterbirds, conserving water, and preventing desertification [18]. In recent years, the construction of upstream reservoirs along the Nenjiang River has significantly reduced water inflow to downstream wetlands, altering their natural hydrological regime. Anthropogenic activities, including agricultural drainage inputs, road construction, ditch drainage, and cofferdam development, coupled with climate-induced warming and drying trends, have substantially altered wetland hydrological regimes and aquatic environments [10,19]. Receiving agricultural drainage water is regarded as an effective measure to replenish the ecological water demand for vegetation and maintain it in wetlands. However, the content of pollutants and salinity in the water has been increased, and the distribution pattern and growth of typical wetland vegetation are severely threatened [20]. Alterations in wetland aquatic environments accelerate vegetation succession processes while increasing the unpredictability of their developmental trajectories. The current status and change trend in wetland vegetation in the context of hydrological and water environment changes need to be comprehensively evaluated. Although existing studies have paid some attention to the responses of plant communities to hydrological and climatic changes [21,22,23], studies on the effects of water environmental changes on N and P stoichiometry of typical wetland plants are relatively limited.

*Bolboschoenus planiculmis* is widely distributed in the typical soda–alkali wetland of the semi-arid area of the Songnen Plain and serves as an important food source for the migratory rare water bird, the white crane (*Grus leucogeranus*). In recent years, anthropogenic disturbances and climate change have drastically transformed the plant community composition and structure of *B. planiculmis* wetlands, resulting in a substantial decline in suitable white crane habitats [10,18]. Studies on vegetation in the *B. planiculmis* wetlands mainly focus on the response of plant communities and populations to water level changes and the ecological adaptation of individual plants to salt and alkali conditions [24,25]. However, the adaptive responses of plant N and P stoichiometry to multiple aquatic environmental stressors remain unexplored. The aim of this study is threefold: (1) to investigate the differences in N and P content and their ratios in leaves, stems, tubers, and roots of *B. planiculmis*; (2) to identify the key water variables governing the N and P stoichiometry of *B. planiculmis* plants; and (3) to clarify the influence path of water variables on the N and P stoichiometry of the *B. planiculmis* plants. The results of this study can provide crucial data for predicting the population stability of *B. planiculmis* under the influence of continuous changes in the water environment caused by agricultural drainage water input in the semi-arid regions.

## 2. Results

### 2.1. Variations in N and P Stoichiometry in Organs of B. planiculmis Plants

The results regarding N content in the organs of *B. planiculmis* indicated that the N content in leaves was the highest at 16.22 g kg^−1^ (*p* < 0.05; Table 1). There was no significant difference in N content among other organs (*p* > 0.05). The tuber had the highest P content at 2.78 g kg^−1^, followed by the leaf, stem, and roots. In terms of the N:P ratio, the leaf had the highest level of 1.2, followed by stem and roots. The tuber had the lowest level at 2.89. Nevertheless, the leaf N:P ratio was less than 14, suggesting that plant growth was more restricted by N limitation.

### 2.2. Relationships of N and P Contents in Organs of B. planiculmis Plants

**Figure 1:**
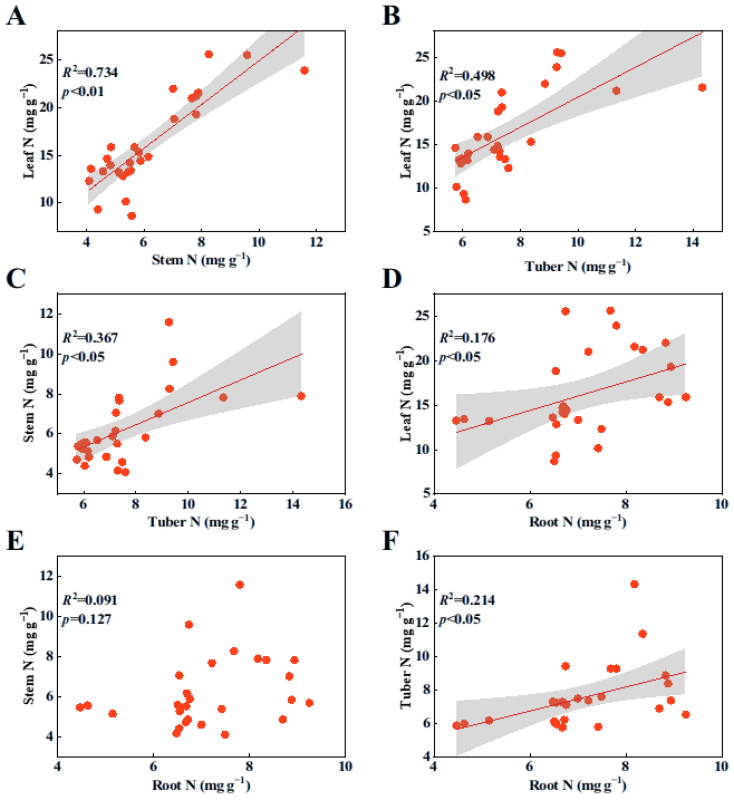
Relationships among N contents in various organs of *B. planiculmis* plants. (**A**), relationship between stem N and leaf N; (**B**), relationship between tuber N and leaf N; (**C**), relationship between tuber N and stem N; (**D**), relationship between root N and leaf N; (**E**), relationship between root N and stem N; and (**F**), relationship between root N and tuber N.

**Figure 2:**
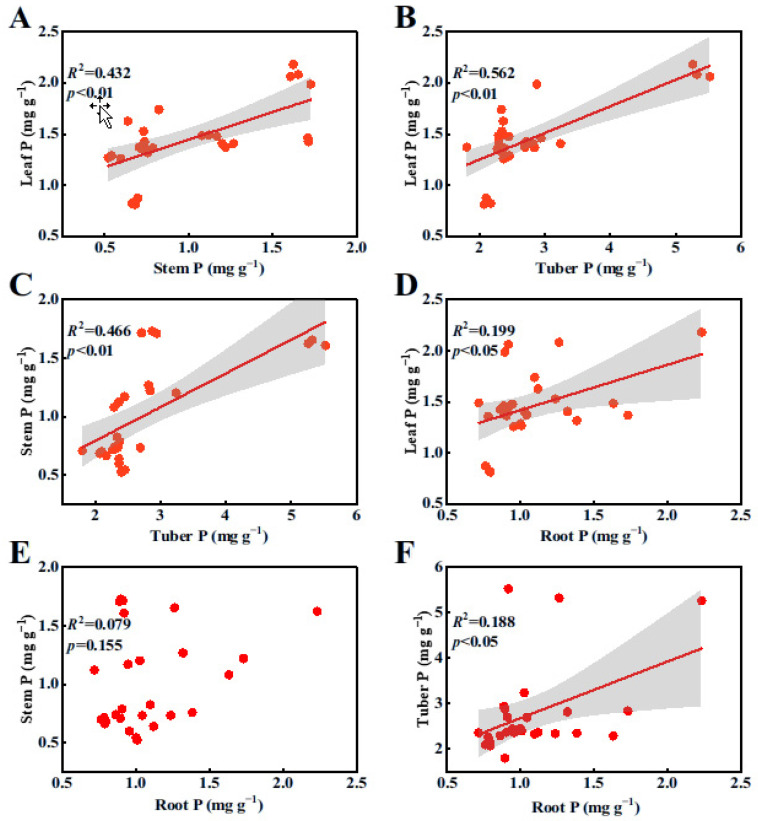
Relationships among P contents in various organs of *B. planiculmis* plants. (**A**), relationship between stem P and leaf P; (**B**), relationship between tuber P and leaf P; (**C**), relationship between tuber P and stem P; (**D**), relationship between root P and leaf P; (**E**), relationship between root P and stem P; and (**F**), relationship between root P and tuber P.

Pearson’s correlation analyses revealed that there was a significant linear relationship among the N contents in each organ (Figure 1A–D,F; *p* < 0.05), with the exception of the relationship between stem N and root N contents (Figure 1E, *p* > 0.05). Similarly, there was a significant positive correlation among the P contents in all plant organs (Figure 2A–D,F; *p* < 0.05), except for the relationship between stem P and root P contents (Figure 2E, *p* > 0.05).

### 2.3. Effects of Water Variables on N, P, and N:P in Organs of B. planiculmis Plants

**Figure 3:**
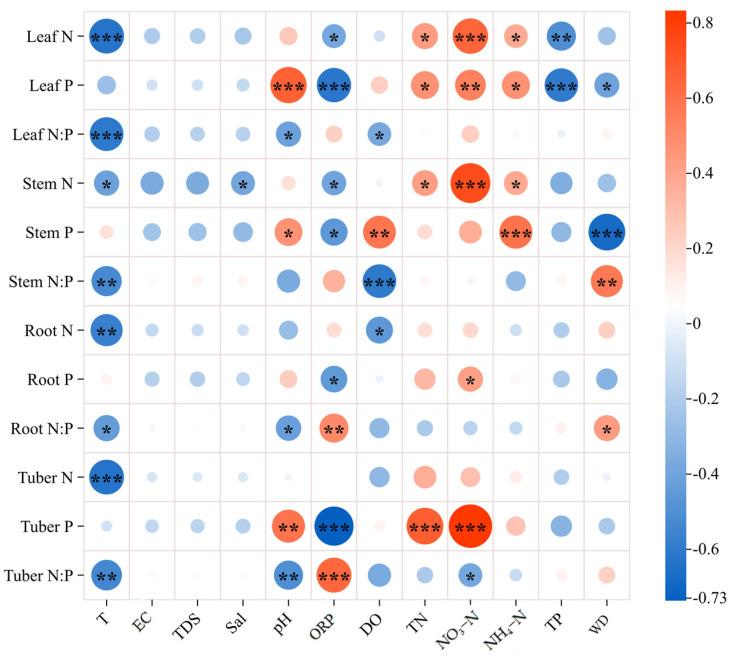
Correlations of N, P, and N:P ratio of *B. planiculmis* organs with water variables. T, water temperature; EC, electrical conductivity in water; TDS, total dissolved solids in water; Sal, salinity of water; ORP; oxidation reduction potential of water; DO, dissolved oxygen in water; TN, total nitrogen content in water; NO_3_–N, nitrate nitrogen content in water; NH_4_–N, ammonium nitrogen content in water; TP, total phosphorus content in water; WD, water depth. ***, *p* < 0.001; **, *p* < 0.01; *, *p* < 0.05.

The relationships in Figure 3 indicate that some water variables had significant effects on N, P, and N:P ratio in organs of the *B. planiculmis* plant, encompassing both positive and negative correlations. The N content in water, such as TN, NH₄–N, and NO₃–N, had significant positive correlations with the N and P contents in *B. planiculmis* organs. However, there was no significant positive correlation with N:P ratio (*p* < 0.05). In fact, there was a negative correlation between NO₃-N and the tuber N:P ratio (*p* < 0.05). Additionally, N and N:P in organs were negatively correlated with water T (*p* < 0.05), but there was no significant relationship between the P content in organs and T (*p* > 0.05). Significant negative correlations were observed between water TP and leaf N and P contents (*p* < 0.05). Water quality parameters (pH, ORP, DO, WD) exhibited both positive and negative correlations with N, P, and N:P ratios across plant organs.

**Figure 4:**
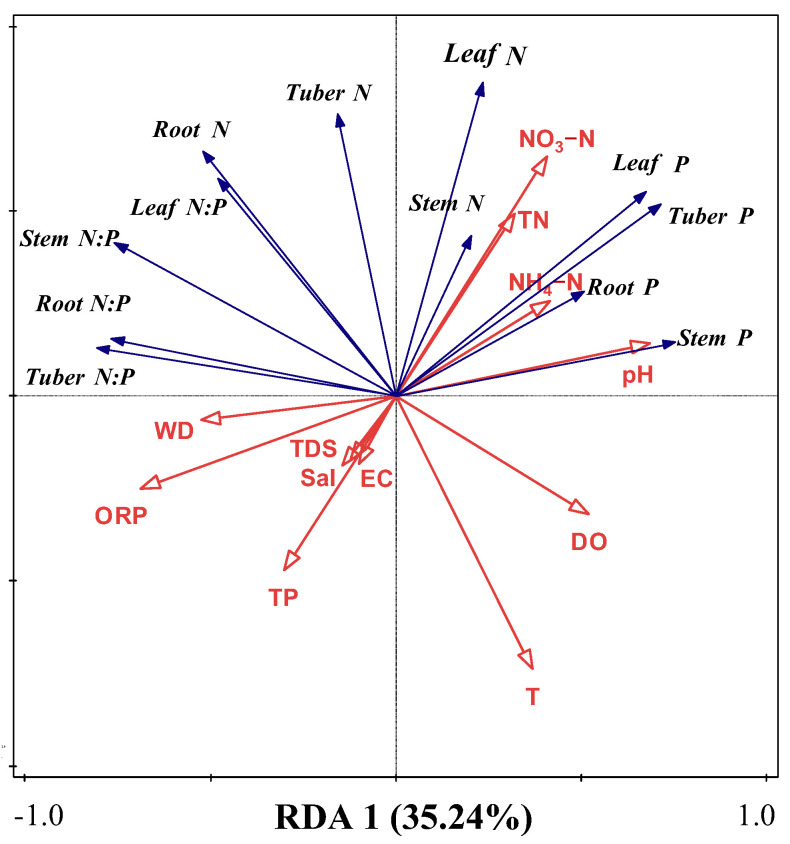
RDA between water environmental parameters and N, P, and N:P ratio of *B. planiculmis*. T, water temperature; DO, dissolved oxygen in water; TN, total nitrogen content in water; NH_4_–N, ammonium nitrogen content in water; NO_3_–N, nitrate nitrogen content in water; TP, total phosphorus content in water; EC, electrical conductivity in water; TDS, total dissolved solids in water; Sal, salinity of water; ORP; oxidation reduction potential of water; WD, water depth.

RDA was used to analyze the effects of water variables on N, P, and N:P ratios across plant organs (Figure 4). Results revealed that overall water variables explained 61.61% (axis 1, 35.24%; axis 2, 26.37%) of the total variations (*F* = 3.1; *p* = 0.002). Conditional effects showed that ORP, NH_4_–N, NO_3_–N, WD, and TN could effectively explain N and P stoichiometry (Table 2), accounting for 19.9% (*F* = 6.2; *p* = 0.002), 18.6% (*F* = 7.3; *p* = 0.002), 11.8% (*F* = 5.5; *p* = 0.002), 8.7% (*F* = 4.7; *p* = 0.002), 5.0% (*F* = 2.9; *p* = 0.022), and 3.5% (*F* = 2.3; *p* = 0.036) of the variations, respectively. These results indicate that these water variables, especially T, N, and WD, were the most important factors affecting N and P stoichiometry.

### 2.4. Influences of Water Variables on N and P Contents and N:P Ratio in Leaves, Stems, Tubers, and Roots

**Figure 5:**
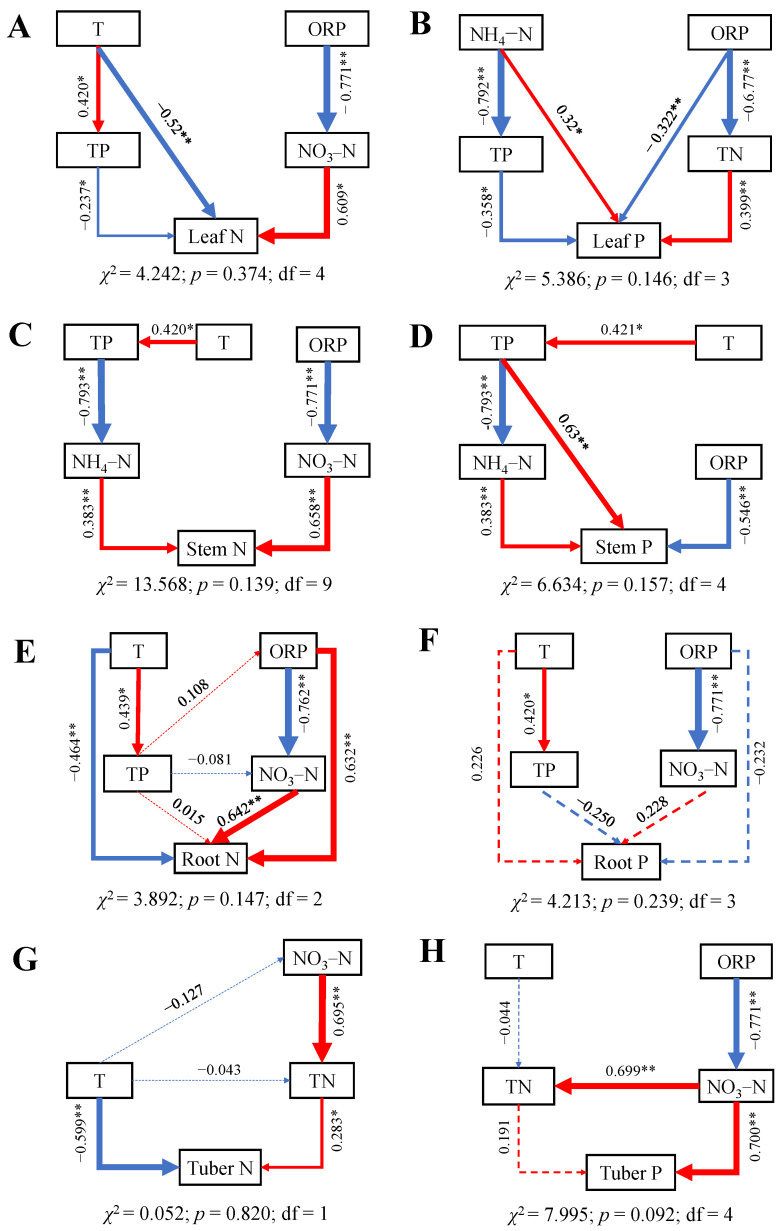
SEMs of water variables on N and P contents of *B. planiculmis* organs. (**A**), SEM for leaf N; (**B**), SEM for leaf P; (**C**), SEM for stem N; (**D**), SEM for stem P; (**E**), SEM for root N; (**F**), SEM for root P; (**G**), SEM for tuber N; and (**H**), SEM for tuber P. Abbreviations are as follows: T, water temperature; TN, total nitrogen content in water; NH_4_–N, ammonium nitrogen content in water; NO_3_–N, nitrate nitrogen content in water; TP, total phosphorus content in water; ORP; oxidation reduction potential of water. **, *p* < 0.01; *, *p* < 0.05.

The results of the SEMs demonstrated that the impacts of water variables on N and P contents in all organs were significant (Figure 5), with nearly all exhibiting significant paths (*p* > 0.05). Water T had a direct negative effect on leaf, root, and tuber N (Figure 5A,E,G), while it had indirect negative effect on stem N (Figure 5C). Additionally, N in water, especially NO_3_–N, had a direct or indirect positive effect on N contents in each organ. ORP and TP also directly or indirectly affect N content in various organs. The content of TP in water had a direct negative effect on leaf P and a direct positive effect on stem P. The NH_4_–N in water directly influenced the accumulation of P in aboveground organs. The effects of various water environmental factors on root P were valid, but no water variables had a direct impact on root P.

**Figure 6:**
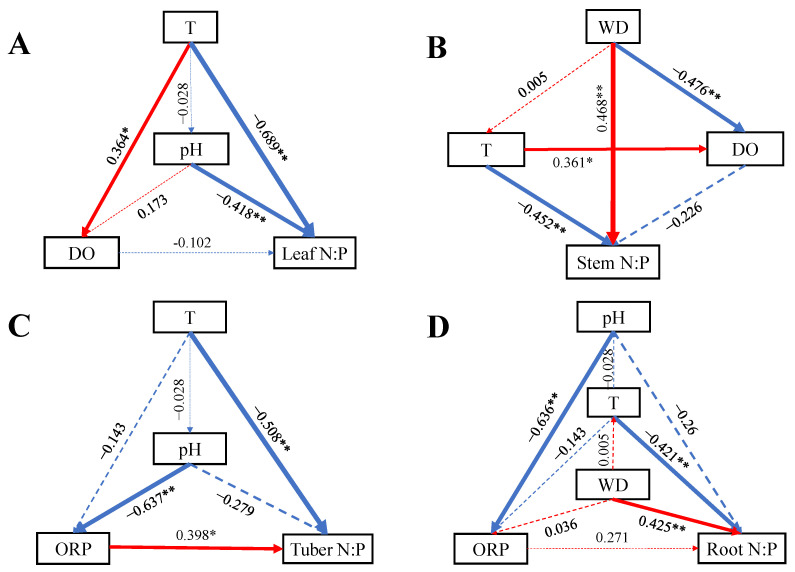
SEMs of water variables on *B. planiculmis* organs N:P (saturated models). (**A**), SEM for leaf N:P; (**B**), SEM for stem N:P; (**C**), SEM for tuber N:P; and (**D**), SEM for root N:P. Abbreviations are as follows: T, water temperature; DO, dissolved oxygen in water; ORP; oxidation reduction potential of water; WD, water depth. **, *p* < 0.01; *, *p* < 0.05.

Based on the results of RDA, some significant variables were incorporated into SEM to analyze their effects on N:P ratios (Figure 6). All SEMs were saturated. Water T had a direct negative effect on N:P ratios in each organ, WD had direct positive effect on stem N:P and root N:P, and pH had direct and indirect effect on leaf and tuber N:P ratios. The ORP had a direct positive effect on tuber N:P ratio.

## 3. Discussion

When the water environment changes, such as with fluctuations in water level and changes in water quality, plants need to adjust their physiological metabolism to adapt to the new environment. The change in the water environment due to agricultural drainage water entering wetlands may impact plants’ absorption and distribution of nutrients [26,27]. This study revealed significant interspecific variation in N content across plant organs, with leaf tissues exhibiting the highest level. This phenomenon primarily stems from aquatic environmental changes exerting survival pressures on plants, with leaf growth and expansion serving as key adaptive strategies to environmental fluctuations. For instance, plants tend to enhance their own adaptability by increasing the N content of leaves [28]. Moreover, due to their location and physiological characteristics, leaves may possess certain advantages in obtaining nutrients such as N. Plants may preferentially allocate N to their leaves to maintain key physiological processes like photosynthesis [29]. Organs like roots and stems are primarily responsible for the absorption and transportation of water and nutrients. Their N requirements are relatively focused on maintaining basic physiological functions and providing structural support [5], consequently exhibiting a lower content than that of leaves. The tubers of *B. planiculmis* play the role of storing nutrients within its body. Under fluctuating aquatic conditions, nutrient availability may become unstable. *B. planiculmis* adapts by allocating essential nutrients, particularly P, to tuber storage for rapid remobilization during resource scarcity. In the case of rising water levels that reduce the availability of P in the soil, plants can obtain P from tubers to maintain growth. Compared with other organs, the growth and metabolism of bulbous rhizomes are relatively slow, and the demand for P is relatively stable [30]. Plants tend to prioritize the allocation of limited P resources to rhizomes to ensure their viability under adverse conditions. This also explains why the P content in *B. planiculmis* tubers was higher than that in leaves, stems, and roots in this study.

The N:P ratio of plant organs can reflect the nutrient restriction status of plants. A high N:P ratio implies that the plant is relatively deficient in P, and its growth is mainly limited by P. Conversely, a lower N:P ratio may indicate that the plant is relatively deficient in N and its growth is restricted by N [31]. In this study, the N:P ratio of *B. planiculmis* leaves was higher than that of other organs; however, it was still less than 14, indicating that plant growth was more limited by N. Agricultural drainage often brings more N into the water. Nitrogen limitation primarily arises from two interconnected factors. On the one hand, agricultural drainage induces soil waterlogging, which compromises soil aeration, thereby impairing root respiration and N uptake efficiency [32,33]. On the other hand, the N in the agricultural drainage water may predominantly exist in forms that are hard for some plants to use; or the N might have chemical imbalances, which may not be appropriate for the growth requirements of plants [15,34,35]. Therefore, changes in the wetland water environment, such as water level changes, water quality deterioration, and salinity increase, will affect the absorption and utilization of N and P by plants, thus altering the N:P ratio in the organs of *B. planiculmis*.

Nutrient allocation across plant organs is a highly dynamic physiological process. In the case of a changing water environment, plants may adjust their resource allocation strategies [36]. In this study, there was a significant positive correlation between N and P in the *B. planiculmis* tubers, stems, and leaves. Plants possess a sophisticated N and P uptake and translocation system that facilitates nutrient transport from roots to aboveground organs [37,38]. When the water environment changes, plants may adjust the distribution and metabolism of N and P according to the growth needs of different organs [39,40], resulting in the N content in each organ maintaining a relatively stable positive correlation. In addition, the relationship between root and stem N and P was not significant. This phenomenon is primarily attributed to the direct yet variable impacts of aquatic environmental changes on root and stem growth and their physiological status. For example, too much water may cause oxygen deprivation to the roots, affecting their N and P uptake and metabolism [41,42]. However, the stem is probably affected by water conduction, gas exchange, and other factors [43]. Other factors in the water environment, such as pH, salinity, and heavy metal content, may also have different effects on roots and stems, and thus affect the relationship between N and P contents [44].

Correlations and RDA results showed that water T, ORP, and WD, as well as N levels— including TN, NH_4_–N, and NO_3_–N—had important effects on the distribution pattern of N and P in *B. planiculmis* plants. Otherwise, RDA indicated that salinity and pH were not the main influencing factors. The main reason is that receiving agricultural drainage water has alleviated drought and saline–alkali conditions to a certain extent [18]. In addition, SEMs also confirmed that these water environment variables also directly and indirectly affected the distribution pattern of N and P in plants. Water T can affect the nutrient absorption capacity of roots, changes the metabolic process in plants, affects plant growth rate and biomass allocation, and then affects the transport and allocation of N and P in roots [45]. Water T levels that are too low or too high may inhibit plant growth, resulting in reduced biomass. In this case, plants may adjust their allocation strategies for N and P. In addition, water T affects the form and availability of N and P in the water body, as well as the solubility and diffusion rate of N and P in the water body, thus affecting the absorption of them by plants. A warmer water T generally increases the rate of respiration, making plants use more energy [45]. This may affect the N and P requirements of plants, which in turn affects the N:P ratio in roots and leaves. This also explains the effect of water T on the N and P contents and their ratios in *B. planiculmis* organs in the SEM. Consequently, water T significantly shapes plant N and P distribution patterns by regulating physiological processes, growth dynamics, and morphological adaptations in wetland plants, while simultaneously influencing environmental parameters. In addition, water ORP had a significant effect on the distribution pattern of N and P in *B. planiculmis* plants, and the change in water ORP likely affected the form of N in water. In the high-ORP environment, N mainly exists in the form of NO_3_–N, which is easily absorbed by plants and has strong mobility in plants [46], and P exists mainly in the form of orthophosphate, which has a high solubility and is easily absorbed by plants [47]. Therefore, the *B. planiculmis* plants in this situation could take up more N and P and distribute it to areas of strong growth, such as leaves and stems.

This study revealed that agricultural drainage-induced water level fluctuations significantly alter *B. planiculmis* N:P stoichiometry. Elevated water levels from agricultural drainage cause soil inundation, creating anaerobic conditions that suppress aerobic microbial activity, thereby inhibiting organic matter decomposition and nutrient mineralization processes [48]. For instance, the mineralization of organic N is reduced, resulting in less NH_4_– and NO_3_–N that can be taken up and utilized by plants. At the same time, the anaerobic environment will also promote some chemical reactions that are not conducive to plant growth, such as an enhancement of denitrification, so that the nitrate in the soil is converted to lost N, further reducing the N available to plants [49]. Furthermore, under anaerobic conditions, metal ions such as iron and aluminum in soil may combine with P to form insoluble compounds, and then reduce P availability [50]. In addition, water level changes can affect the growth and distribution of wetland plant roots, as well as photosynthesis [51]. Therefore, water level fluctuations directly impact both soil conditions and root development, subsequently influencing nutrient acquisition. Simultaneously, root systems adapt to water level variations through morphological and physiological adjustments, thereby modulating N and P uptake and allocation patterns.

When the contents of N and P in agricultural drainage water are high, wetland plants will absorb a large amount of N and P, resulting in a significant increase in the content of these nutrients in plants [15]. This may disrupt the balance of N and P in plants and affect the normal growth and metabolism of plants [52], as there is a certain correlation between N and P metabolism in plants. A high N content in water may cause plants to distribute more N to the aboveground portion to promote photosynthesis and growth [53]. Simultaneously, a high N content may also inhibit the uptake of P by plants or cause plants to distribute more P to the roots to maintain the normal metabolic function of the roots [54,55]. Therefore, the SEM results of this study indicated that the P content in the tubers, stems, and leaves was also directly affected by the N content in water. In addition, a high level of N tends to affect hormone levels and signaling in plants, further regulating the distribution pattern of N and P in plants [56]. Both the RDA and SEM results of this study identified TN, NH_4_–N, and NO_3_–N as key environmental drivers of N and P distribution in *B. planiculmis*.

## 4. Materials and Methods

### 4.1. Study Area

The study was conducted in the Momoge wetlands (45°28′–46°18′ N, 122°47′–124°43′ E), situated on the western bank of the Nenjiang River within China’s Songnen Plain. This area occupies 1440 km^2^, with a wetland area of 1040 km^2^. The wetland develops in the lakeside depression, flood plain, and low depression. The water source is supplied by Nenjiang River tributaries, surface water, and atmospheric precipitation. In this area, the climate is a continental monsoon climate. In spring, it is characterized by drought and scant rainfall, accompanied by a rapid rise in temperature. Summer is swelteringly hot, with rainfall concentrated from July to September. Autumn witnesses a sharp drop in temperature and is mostly sunny. Winter is extremely cold with little snowfall. The average annual temperature stands at 4.4 °C. The average annual precipitation is 390 mm, while the average annual evaporation amounts to 1472 mm.

The *B. planiculmis* communities are mainly distributed in the central and western parts of Momoge wetlands, providing a food source and habitat for rare water birds such as white cranes. The associated species of *B. planiculmis* communities in the wetlands located in the transition area of water and land are *Suaeda salsa*, *Setaria viridis*, *Leymus chinensis*, *Puccinellia tenuiflora*, and *Poa* spp. In flooded areas, there is often a single population, or one associated with *Phragmites australis*, *Scirpus validus*, and *Typha angustifolia*. At present, the wetlands are undergoing an agricultural drainage water input, which has changed the water environment, and also affected the dynamics of the *B. planiculmis* community.

### 4.2. Field Survey and Sampling

**Figure 7:**
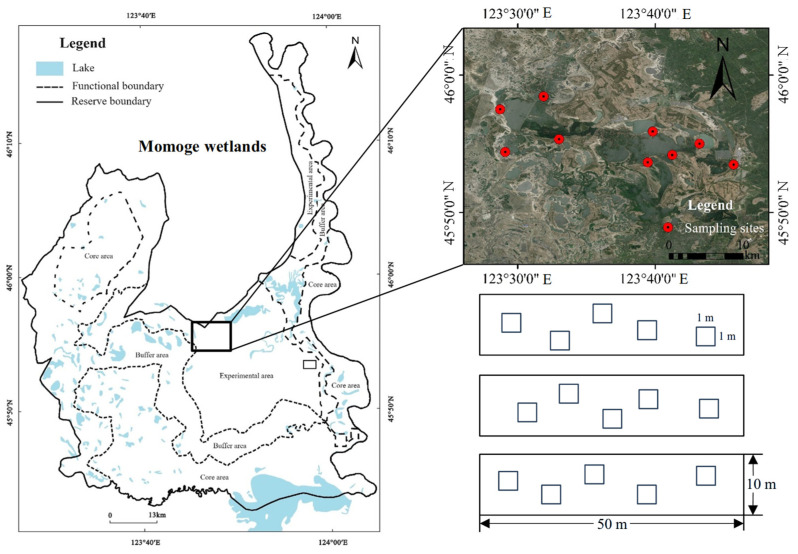
Schematic diagram of study area, sampling sites, and experimental design.

In this study, we selected 10 *B. planiculmis* wetland sites in the middle of the Momoge wetlands as the research area (Figure 7). These sites were flooded and mainly dominated by *B. planiculmis* populations. The method of combining a transect and quadrat was adopted, and 3 transects (50 m length × 10 m width) at a distance of >20 m were set at each wetland site, and five 1 m × 1 m quadrats were randomly set along the transects. Starting from 10 May 2021, the water depth was recorded in each quadrat every two weeks, and a portable multi-parameter water quality meter (YSI ProQuatro, Columbus, OH, USA) was used to measure the water temperature (T, °C); pH; dissolved oxygen (DO, mg⋅L^−1^); electrical conductivity (EC, ms cm^−1^); total dissolved solids including inorganic salts, organics, and ions (TDS, mg⋅L^−1^); salinity (Sal, ppt); and oxidation reduction potential (ORP, mv). At the same time, wetland water samples were collected and brought back to the laboratory for the determination of total nitrogen content (TN, mg⋅L^−1^), ammonium nitrogen content (NH_4_–N, mg⋅L^−1^), nitrate nitrogen content (NO_3_–N, mg⋅L^−1^), and total P content (TP, mg⋅L^−1^). TN in water was determined by ultraviolet spectrophotometry with potassium persulfate digestion. NH_4_–N was determined by spectrophotometry with Nesser’s reagent. NO_3_–N was determined by sulfamic acid ultraviolet spectrophotometry. TP in water was determined by ammonium molybdate spectrophotometry. The water quality was measured until the end of August, and then averaged to represent the various water conditions.

At the end of August, according to the above transects and quadrats, the aboveground part of *B. planiculmis* was collected, and the stems and leaves were separated. Root samples within 0–30 cm soil depth were collected and washed with clean water. Afterward, the root tubers were separated and counted. The stem, leaf, root, and tuber samples were placed in a drying oven at 65 °C to reach a constant weight. The contents of N and P were determined after crushing. Plant N was determined using the micro-Kjeldahl method. Plant P was determined using the Vanadate–Molybdate–Yellow colorimetry method. Nitrogen to phosphorus (N:P) ratio was obtained by dividing N content by P content.

### 4.3. Data Analysis

Data were analyzed by SPSS 16.0, Canoco 5.0, and R v3.4.2. One-way analyses of variance (ANOVAs) were employed to explore the differences in N and P contents among plant leaves, stems, tubers, and roots at a significance level of *p* ≤ 0.05. Pearson’s correlation analyses were used to explore correlations between N and P contents among plant organs and water variables at *p* ≤ 0.05, *p* ≤ 0.01, and *p* ≤ 0.001 levels. A redundancy analysis (RDA) was employed to identify the main water variables that affect the N and P stoichiometry in plants. Structural equation models (SEMs) were used to evaluate the influencing pathways of water variables on N and P stoichiometry in plants. Prior to the SEM procedure, we reduced the number of water variables based on the results of RDA while considering the collinearity among variables. The chi-squared test (*χ*^2^) and *p*-value were used to evaluate the model fitness.

## 5. Conclusions

The wetlands in semi-arid regions currently face the dual stressors of drought and saline alkalization. While agricultural drainage water supplementation partially mitigates water scarcity, it significantly alters plant N and P stoichiometry. Firstly, there were significant differences in N, P, and the N:P ratio among *B. planiculmis* organs. Secondly, there were significant positive correlations between the N:P contents in all organs, except for the relationship between roots and stems. Thirdly, RDA indicated that water T, ORP, N contents, and WD are the main factors affecting N and P stoichiometry. SEMs further demonstrated the direct and indirect effects of these water variables. This study demonstrated that agricultural drainage water inputs mitigated wetland water scarcity and saline–alkali constraints on plant nutrient dynamics, while simultaneously amplifying the influence of key water variables (T, WD, N, and ORP) on plant N and P stoichiometry. Therefore, wetland vegetation and white crane habitat restoration in arid/semi-arid regions requires integrated consideration of hydrological regimes, water quality parameters, and water source characteristics.

## Figures and Tables

**Table 1:** Contents of N, P, and their ratios in organs of *B. planiculmis* plants.

	Leaf		Stem		Tuber		Root			
Nutrient	Mean ± SE	CV (%)	Mean ± SE	CV (%)	Mean ± SE	CV (%)	Mean ± SE	CV (%)	*F*	*p*
N (g kg^−1^)	16.22 ± 4.62 a	28.47	6.21 ± 1.74 b	27.95	7.55 ± 1.71 b	22.70	7.14 ± 1.13 b	15.80	75.976	<0.001
P (g kg^−1^)	1.45 ± 0.32 b	22.04	1.02 ± 0.41 c	40.07	2.78 ± 0.95 a	34.26	1.08 ± 0.21 c	19.51	53.155	<0.001
N:P	11.2 ± 2.16 a	19.28	6.71 ± 2.02 b	30.04	2.89 ± 0.90 c	31.26	7.07 ± 1.94 b	27.48	90.322	<0.001

Notes: different lowercase letters represent significant differences in each organ at *p* ≤ 0.05 level. SE, standard error; CV, coefficient of variation.

**Table 2:** Simple and conditional effects of water variables on N, P, and N:P ratio of *B. planiculmis*.

Variables	Simple Effects (Explains%)	*F*	*p*	Conditional Effects (Explains%)	*F*	*p*
T	19.9	6.2	0.002	19.9	6.2	0.002
ORP	19.1	5.9	0.002	18.6	7.3	0.002
NH_4_–N	9.6	2.7	0.028	11.8	5.5	0.002
NO_3_–N	18.1	5.5	0.004	8.7	4.7	0.002
WD	11.6	3.3	0.010	5.0	2.9	0.022
TN	10.9	3.1	0.022	3.5	2.3	0.036
pH	17.8	5.4	0.004	0.9	0.7	0.592
DO	13.3	3.8	0.014	2.8	1.9	0.116
TP	9.7	2.7	0.032	3.3	2.0	0.066
Sal	2.1	0.5	0.714	1.0	0.7	0.594
TDS	1.8	0.5	0.804	3.0	2.2	0.076
EC	1.7	0.4	0.808	2.8	2.2	0.084

Notes: Abbreviations are as follows: T, water temperature; ORP; oxidation reduction potential of water; NH_4_–N, ammonium nitrogen content in water; NO_3_–N, nitrate nitrogen content in water; WD, water depth; TN, total nitrogen content in water; DO, dissolved oxygen in water; TP, total phosphorus content in water; Sal, salinity of water; TDS, total dissolved solids in water; EC, electrical conductivity in water.

## Data Availability

The original contributions presented in this study are included in the article; further inquiries can be directed to the corresponding author.
